# Brazilian Protocol for Sexually Transmitted Infections, 2020: congenital syphilis and child exposed to syphilis

**DOI:** 10.1590/0037-8682-597-2020

**Published:** 2021-05-17

**Authors:** Carmen Silvia Bruniera Domingues, Geraldo Duarte, Mauro Romero Leal Passos, Denise Cardoso das Neves Sztajnbok, Maria Luiza Bezerra Menezes

**Affiliations:** 1 Secretaria de Estado da Saúde de São Paulo, Centro de Referência e Treinamento de Doenças Sexualmente Transmissíveis e Aids, São Paulo, SP, Brazil.; 2 Universidade de São Paulo, Faculdade de Medicina de Ribeirão Preto, Ribeirão Preto, SP, Brazil.; 3 Universidade Federal Fluminense, Departamento de Microbiologia e Parasitologia, Niterói, RJ, Brazil.; 4 Universidade do Estado do Rio de Janeiro, Faculdade de Ciências Médicas, Rio de Janeiro, RJ, Brazil.; 5 Universidade de Pernambuco, Departamento Materno-Infantil, Recife, PE, Brazil.

**Keywords:** Syphilis, Congenital syphilis, Prenatal care, Diagnosis, Therapeutics, Monitoring

## Abstract

The topics of congenital syphilis and children exposed to syphilis compose the Clinical Protocol and Therapeutic Guidelines for Comprehensive Care for People with Sexually Transmitted Infections, published by the Brazilian Ministry of Health in 2020. Such document was elaborated based on scientific evidence and validated in discussions with specialists. This article provides guidelines for syphilis in pregnant women and congenital syphilis clinical management, emphasizing the vertical transmission of *Treponema pallidum* prevention. Epidemiological and clinical aspects of these infections are presented and recommendations for managers in the programmatic and operational management of syphilis. The article also includes guidelines for health professionals in screening, diagnosing, and treating people with sexually transmitted infections and their sex partners, in addition to strategies for surveillance actions, prevention, and control of the disease.

## Highlighted excerpt

Most congenital syphilis cases arise from test failures in prenatal care or inadequate or no treatment of maternal syphilis.

## FOREWORD

This article addresses congenital syphilis and child exposed to syphilis topics, present in the Clinical Protocol and Therapeutic Guidelines (PDCT) for Comprehensive Care for People with Sexually Transmitted Infections (STI), published by the Health Surveillance Department of the Brazilian Ministry of Health. For elaborating the PDCT, a selection and analysis of the evidence available in the literature were performed, and a panel with specialists discussed it. The PDCT was approved by the National Committee for the Incorporation of Technologies to the Brazilian National Health System (Conitec) and updated by the team of specialists in STI in 2020^1^.

## EPIDEMIOLOGICAL ASPECTS

In 2016, there was an estimate of 661,000 congenital syphilis cases worldwide^2^. In Brazil, for the period 2014-2018, the number of acquired syphilis in adults increased and syphilis in pregnant women and congenital syphilis^3^. Such increase can be attributed to a more extensive testing number, arising from the dissemination of rapid tests and the decrease in condom use, penicillin administration in primary health care services reduction, and the shortage of this medicine worldwide^4^. For the period 2010-2019 (data up to June 30, 2019), Brazil registered 650,258 acquired syphilis cases, 297,003 syphilis in pregnant women 162,173 congenital syphilis cases. In the same period, 11,480 early and late fetal deaths were reported as attributed to congenital syphilis^3^. 

Congenital syphilis is an avoidable disease, provided that syphilis in pregnancy is diagnosed and treated adequately. However, despite the efforts, it still is a serious public health issue, and it shows failures, especially in prenatal care. Most congenital syphilis cases arise from test failures in prenatal care or inadequate or no treatment of maternal syphilis^5^ ^-^ ^7^. It results from *Treponema pallidum* hematogenic dissemination by the non-treated or inadequately treated infected pregnant woman to her conceptus, generally through transplacental route, regardless of the pregnancy stage^4^.This transmission can occur during birth, through direct contact with syphilitic lesions in the birth canal^8^ ^-^ ^10^. 

The transplacental passing of treponemas during pregnancy may occur in any clinical stage of maternal syphilis. However, vertical transmission is more frequent in recent syphilis (primary lesions, secondary lesions, and recent latent syphilis up to one year), decreasing with the disease’s evolution to late phases (late latent after one year and late in tertiary syphilis)^11^. The reduction in transmission probability is directly associated with the decrease of circulating treponemas, ranging from 70% to 100% in syphilis with primary or secondary lesions to 30% in recent latent or late syphilis^6^ ^,^ ^12^. In addition to being characterized by higher transmissibility, the recent maternal syphilis phase can severely affect the fetus^13^. Circulating treponemas charge drops, but it does not disappear if there is no adequate treatment. Additionally to the syphilis clinical stages, vertical transmission occurrence is also influenced by the amount of time the fetus is exposed^4^. 

Among the adverse outcomes of non-treated maternal syphilis, 40% will lead to early pregnancy loss, 11% to fetal death, and 12% to 13% to preterm birth or low birth weight^2^ ^,^ ^14^. At least 20% of newborns will present signals suggesting congenital syphilis^2^ ^,^ ^12^ ^,^ ^15^. 

## CLINICAL ASPECTS

Congenital syphilis is a disease with a broad clinical specter, and it can manifest itself from asymptomatic or oligosymptomatic forms to severe forms, with septic clinical pictures, fetal and neonatal death. At birth, around 60% to 90% of newborns with congenital syphilis are asymptomatic^16^ ^,^ ^17^, and, for this reason, serological screening of pregnant women in maternity wards is essential. Clinical manifestations of children with congenital syphilis can happen at any moment before they are two years old, generally in the neonatal period. Around two-thirds of children develop symptoms in three to eight weeks, and clinical manifestations are rare after three to four months^18^. 

Congenital syphilis can be didactically divided in early, rising to the second year of life, and late, when the signals and symptoms are observed from the second year of life. In cases of early congenital syphilis, the presence of signals and symptoms at birth depends on the moment of intrauterine infection and the treatment during pregnancy^19^. The following are frequent manifestations of early congenital syphilis: hepatomegaly, splenomegaly, jaundice, serosanguineous rhinitis, maculopapular skin eruptions, syphilitic pemphigus (mainly palmoplantar), generalized lymphadenopathy, bone abnormalities (periostitis, osteochondritis), thrombocytopenia, and anemia. Prematurity and low birth weight are frequent perinatal complications^17^ ^,^ ^20^. 

Late congenital syphilis clinical manifestations are associated with scarring or persistent inflammation of the early infection and are characterized by syphilitic gummas in various tissues. Such manifestations arise in approximately 40% of infected and non-treated children in the first months of life. Some manifestations can be prevented through maternal treatment during pregnancy or child treatment within the first three months of life^21^. However, others, such as interstitial keratitis, Clutton joints, and sensorineural hearing loss, can occur and evolve, despite adequate treatment^22^. In late congenital syphilis cases, the possibility of acquired syphilis arising from child sexual harassment or aggression must be dismissed.

Most mentioned late congenital syphilis manifestations are: Parrot frontal bossing, saddle-nose, ogival palate, interstitial keratitis, chorioretinitis, sensorineural hearing loss, Hutchinson teeth, mulberry molars, developmental delay, intellectual impairment, and saber tibia^19^. 

Congenital syphilis clinical alterations, after *T. pallidum* is released directly into fetal blood, vary and result from an inflammatory response caused by the wide dissemination of spirochaetes in almost every organ and system. Consequently, supplementary examinations are needed to investigate and identify such alterations^19^, such as a complete blood count, transaminases, chest radiography, long bone radiographs, fluid examination, and neuroimaging, when needed^4^.

The central nervous system infection or neurosyphilis can be asymptomatic or symptomatic, occurring in around 60% of the children with congenital syphilis. The following are considered fluid alterations: reactivity in venereal disease research laboratory (VDRL), pleocytosis, and increase in proteinorrachy^21^. During the neonatal period, the following situations are considered neurosyphilis: reacting VDRL in fluid or white blood cells higher than 25 cells/mm³ or protein higher than 150mg/dL, and, in post-neonatal, reacting VDRL in fluid or white blood cells higher than five cell/mm³ or protein higher than 40mg/dL^23^. For the adequate assessment of such values, the fluid must be free from any blood contamination, which can occur in puncture accident cases.

## DIAGNOSTIC

The etiological diagnosis of acquired syphilis requires a correlation between clinical data, laboratory testing results, past investigation history duly registered in medical records, and recent exposure investigation. Only the combination of all information allows for the correct diagnostic evaluation of each case and adequate treatment.

The tests used for diagnosing syphilis are divided into two categories: direct and immunological. Direct examinations include searching for *T. pallidum* in samples collected from lesions, using dark-field microscopy, silver impregnation, immunofluorescence, or polymerase chain reaction molecular biology techniques. The immunological tests, treponemal tests (TT), and non-treponemal test (NTT) are the most used, and they are characterized by the search for antibodies in serum, plasma, fluid, or whole blood samples^4^. 

**FIGURE 1:**
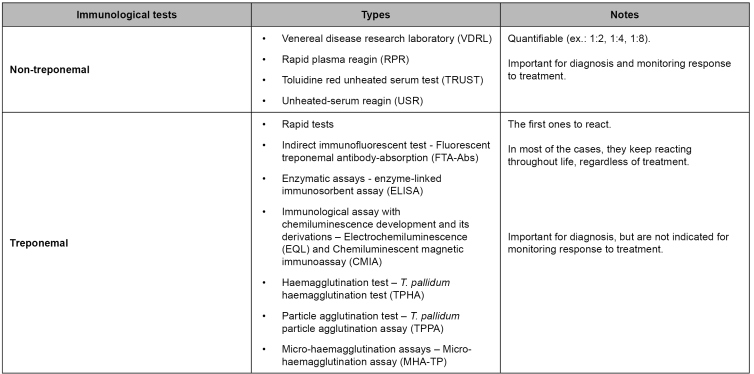
Immunological test used for syphilis diagnosis*.*

**Source:** Clinical Protocol and Therapeutic Guidelines for Comprehensive Care for People with Sexually Transmitted Infections 2020^4^.

TT detects specific antibodies produced against antigens of *T. pallidum*, and NTT detects nonspecific antibodies for antigens of *T. pallidum* (anticardiolipin)^4^. It should be stressed that the rapid tests are TT. Immunological tests used for diagnosing syphilis are presented in Figure 1.

Pregnant women must be tested for syphilis, at least, in the first prenatal care appointment, at the start of the first trimester, and when hospitalized for giving birth. Women presenting early or late pregnancy loss must also be tested and those who have undergone situations of exposure to risk or sexual violence^24^. Considering the sensitivity of the diagnostic flows, starting the investigation with TT, if possible, rapid tests, is recommended. The combination of sequential tests increases the positive predictive value of the reacting result in the initial test^4^. Pregnant women with reacting rapid tests for syphilis must be considered patients with syphilis up to contrary evidence, and, in case of lack of adequate and documented treatment, they must be treated in prenatal care appointment, birth, or miscarriage. At this moment, a confirmation NTT test must be requested and collected (baseline) for monitoring response to treatment^4^. Treatment delay due to waiting for the supplementary test result causes the professional to waste time and opportunity to prevent a congenital syphilis case. The role of good documentation of clinical examination and laboratory examinations and treatments in these women's medical records for the adequate management of syphilis cases should be stressed. 

Initial assessment for children exposed to syphilis must be carried out moreover at maternity hospitals or birth houses, considering maternal history on syphilis regarding treatment and follow-up in pregnancy, clinical signals and symptoms of the child (frequently lacking or nonspecific), and NTT of the child's peripheral blood, simultaneously comparing maternal NTT at the moment of birth. The umbilical cord blood should not be used, as these fetal blood samples can be contaminated by maternal blood and lead to false-reacting results^4^. 

There is no supplementary assessment determining the infection diagnosis in children precisely. Therefore, a combination of clinical, epidemiological, and laboratory assessment is needed^25^. From this perspective, it is crucial to adequately distinguish exposed children (but not infected) from children with congenital syphilis in order to avoid unnecessary conducts, such as invasive examinations and long-lasting hospitalizations^4^. 

For excluding congenital infection in a child exposed to *T. pallidum*, the mother must fulfill all criteria for adequate treatment, with confirmation in medical records or pregnancy notebook, and the examination of the newborn must be normal. The discovery of any signal or symptom must lead to a supplementary investigation for diagnostic confirmation. In addition to physical examination, NTT must be performed at birth for every child exposed to syphilis^4^. 

Simultaneously testing mother and newborn, at immediate postpartum, with the same type of NTT, contributes to determining the meaning of the child's serological findings. A titration higher than that of the mother in at least two dilutions (for example, mother's NTT of 1:4 and newborn's NTT higher than 1:16) indicates congenital infection. Notwithstanding, the lack of this finding does not exclude the possibility of congenital syphilis diagnosis. Some pair studies of mothers with syphilis and newborns showed that less than 30% of children with congenital syphilis present NTT titration four times higher than maternal ones^26^ ^,^ ^27^. Therefore, it is crucial to conduct a detailed physical examination and follow-up of all children.

TT can detect IgM antibodies against *T. pallidum* in the newborn's blood, and such antibodies do not cross the placenta barrier. Therefore, when present in a child sample, they indicate immune system response to syphilis and not a transfer of maternal antibodies. However, the sensitivity of tests based on IgM antibodies is low, and a negative result does not exclude the diagnosis of syphilis in the newborn. For this reason, the use of tests detecting IgM, such as the IgM fluorescent treponemal antibody absorption test and the IgM immunoassays for diagnosing congenital syphilis, is not recommended^4^ ^,^ ^11^ ^,^ ^28^. 

In case of any of the following situations, the child must be deemed a congenital syphilis case and must be notified, investigated, immediately treated, and followed-up regarding clinical and laboratory aspects: a) mother with syphilis inadequately treated or not treated, regardless of clinical assessment or supplementary examinations results on the newborn; b) clinical manifestation present or change in fluid or radiology and reacting NTT, regardless of maternal treatment history and NTT titration; c) newborn's NTT higher than maternal one in at least two dilutions, regardless of the mother's treatment history; and d) persistence of reacting NTT after six months, or reacting TT after 18 months, without previous treatment. It is stressed that all children with congenital syphilis must undergo a complete investigation, including lumbar puncture, to analyze fluid and long bone radiography^4^.

As the congenital syphilis manifestations are nonspecific, the differential diagnosis with other congenital infections, such as toxoplasmosis, rubella, cytomegalovirus, herpes simplex virus, and Zika virus, as well as neonatal sepsis, neonatal hepatitis, and fetal hydrops, is needed, mainly when the newborn's NTT does not react^4^. 

## TREATMENT

**FIGURE 2:**
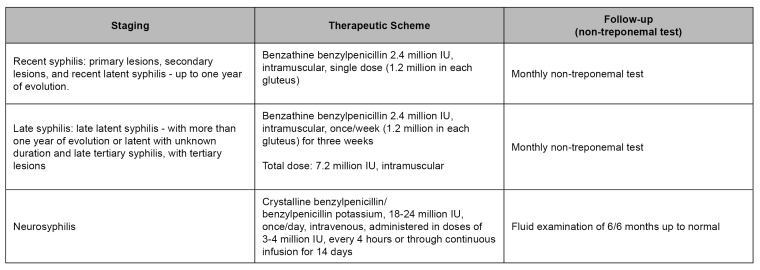
Treatment and follow-up of syphilis in pregnant women*.*

**Source:** adapted from Clinical Protocol and Therapeutic Guidelines for Comprehensive Care for People with Sexually Transmitted Infections, 2020^4^.

**Syphilis in pregnancy:** Benzathine benzylpenicillin is the only medicine that effectively treats pregnant women with syphilis and the fetus, as it crosses the transplacental barrier (Figure 2). Penicillin administration may be performed in private or primary health care services, including in primary health care units^29^,by physicians, nurses^30^ or pharmaceuticals. Anaphylactic reaction to benzathine benzylpenicillin is a rare event, which can take place in around 0.002% of cases^31^ ^,^ ^32^. The seven-day interval between the doses must be kept and monitored to avoid losses during treatment. Healthcare units must actively look for absent pregnant women in order to complete the therapeutic scheme.

Treatments performed with medicines different from benzathine benzylpenicillin during pregnancy are deemed inadequate to avoid vertical transmission. Moreover, maternal treatments are considered adequate only if started before 30 days of birth, and the clinical stage's complete cycle is administered. Pregnant women not fulfilling these criteria will be considered inadequately treated. In turn, newborns will be classified as congenital syphilis cases and undergo clinical, laboratory, and therapeutic assessment and epidemiological notification^4^ (Figure 2). It is essential to highlight that, by the end of 2017, the treatment of the sex partners was taken out from the criterion defining adequate maternal treatment^33^.

Syphilis in pregnant women must be considered a fetal emergency due to the high proportion of early and late fetal deaths associated with lack of treatment. The treatment must be started immediately, even in asymptomatic pregnant women, after syphilis reagent test (TT or NTT). Notwithstanding, setting treatment with only one reacting test does not exclude the need for a second test for a better diagnostic analysis, laboratory monitoring (cure control), and sex partners' treatment^4^.

Jarisch-Herxheimer reaction is an event that can take place in the first 24 hours after the first penicillin dose, mainly in primary and secondary syphilis. It is characterized by exacerbating skin lesions, general discomfort, fever, headache, and arthralgia, which spontaneously decreases after 12 to 24 hours. Mostly in pregnant women treated in the second half of pregnancy, such reaction can lead to preterm birth^11^ ^,^ ^24^. The decrease in syphilis signals and symptoms after treatment indicates a response to therapy. However, the serological follow-up with post-treatment NTT must be monthly up to the end of pregnancy to assess the immune response. After birth, the trimester follow-up must continue until the 12^th^ month after syphilis diagnosis^4^ ^,^ ^34^. The follow-up with the same NTT in the diagnosis is suggested (Figure 1) to avoid divergent titration between the different types - VDRL or rapid plasma reagin test^4^.

Treatment success is traditionally considered as the NTT titration decrease in two dilutions (for example, 1:64 to 1:16) up to three months, after the last penicillin dose, and four dilutions (for example, 1:64 to 1:4) up to six months, with evolution up to seroreversion (non-reacting NTT)^35^. In patients with HIV, in addition to non-reacting NTT, the titration drop in two dilutions up to six months for recent syphilis or up to 12 months for late syphilis can be considered adequate immune response^11^. 

The persistence of low and stable NTT titration after adequate treatment, with previous titration drop in at least two dilutions, is called "serological scar" if new exposures during the analyzed period are discarded. The serological scar does not characterize a therapy failure^4^ ^,^ ^11^. Notwithstanding, NTT with increasing or persistently high titration can indicate reinfection, therapy failure, or neurosyphilis, and the new treatment must be considered^11^. In this situation, the examination performance assessment needs to be evaluated, as it depends on technique, and a serological test for HIV must be conducted. 

It should be highlighted that pregnancy length may not be sufficient to drop two or more NTT titration after treatment. Pregnant women with low titration (for example, 1:2 or 1:4) may not present a drop of more than two titrations or non-reacting NTT result even after birth. Therefore, what is most important in this situation is to rule out the possibility of reinfection and to keep monitoring with NTT.

The following are independent retreatment criteria: a) lack of titration reduction in two dilutions within six months (recent syphilis) or 12 months (late syphilis) after adequate treatment; b) titration increase in two or more dilutions; and c) persistence or recurrence of clinical signals and symptoms^4^. 

The retreatment scheme will depend on the pregnant woman’s disease phase. Neurosyphilis investigation through lumbar (fluid) puncture is also recommended for pregnant women in therapeutic failure when there is no sexual exposure within the period to justify reinfection. For people living with HIV, investigation in all retreatment cases, regardless of recent exposure, is indicated. 

The pregnant woman's sex partners' assessment and treatment are crucial for discontinuing the infection transmission chain. One third the individuals with recent syphilis’ sex partners will develop syphilis within 30 days from exposure^4^. Therefore, in addition to the clinical assessment and laboratory follow-up, in case there is exposure to a person with syphilis within 90 days, presumptive treatment of such partners is recommended, regardless of clinical stage or presence of signals and symptoms, with a single dose of benzathine benzylpenicillin (2.4 million IU, intramuscular) and laboratory testing. If the test is reacting (TT or NTT), treatment as per the clinical stage is recommended^4^.

**FIGURE 3:**
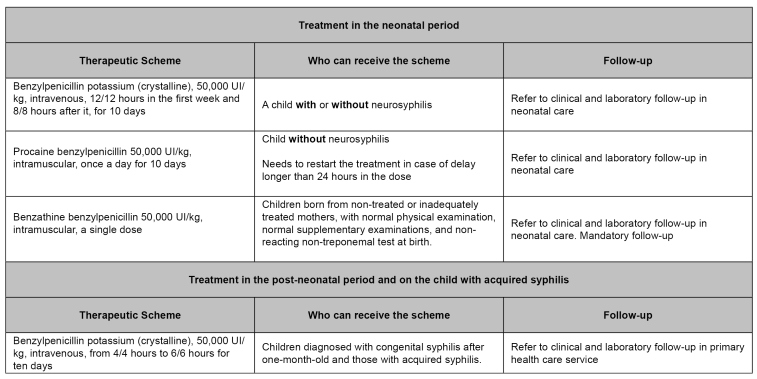
Therapeutic scheme for congenital syphilis in the neonatal period, post-neonatal period, and for children with acquired syphilis.

**Source:** adapted from Clinical Protocol and Therapeutic Guidelines for Comprehensive Care for People with Sexually Transmitted Infections,2020^4^.

**FIGURE 4:**
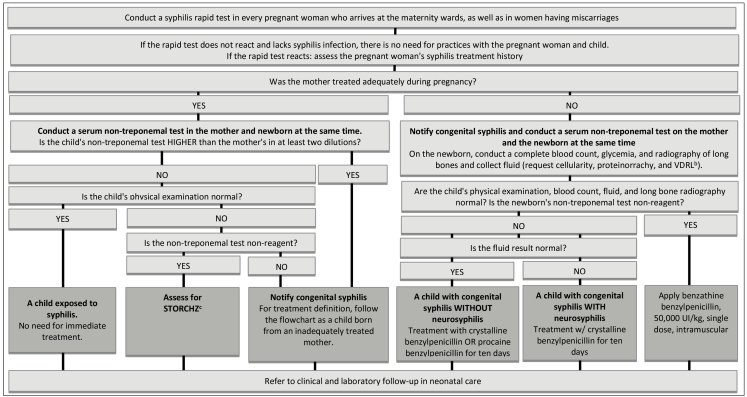
Recommendations for assessment and management in maternity wards of children born from mothers with syphilis diagnosis in current pregnancy or at birth.

**Source:** Clinical Protocol and Therapeutic Guidelines for Comprehensive Care for People with Sexually Transmitted Infections 2020^4^.

**Congenital syphilis and child exposed to syphilis:** Congenital syphilis treatment in the neonatal period is conducted with benzylpenicillin (potassium/crystalline, procaine, or benzathine), depending on the maternal treatment during pregnancy, the newborn's NTT titration in comparison with the mother, and the child's clinical and laboratory examinations. Diagnosed congenital syphilis cases after one month of age (post-neonatal period) and those with acquired syphilis must be treated with crystalline benzylpenicillin or benzylpenicillin potassium (Figure 3 and Figure 4).The complete ten-day scheme with crystalline or procaine benzylpenicillin or benzylpenicillin potassium must be administered even when the child receives ampicillin for other causes.

**Notes:**

a) Conduct a non-treponemal test in peripheral blood of every newborn from mother with the rapid test or reacting non-treponemal test at the moment of birth, regardless of previous treatment.

b) VDRL: venereal disease research laboratory.

c) STORCHZ: syphilis, toxoplasmosis, rubella, cytomegalovirus, herpes simplex virus, and Zika virus.

**FIGURE 5:**
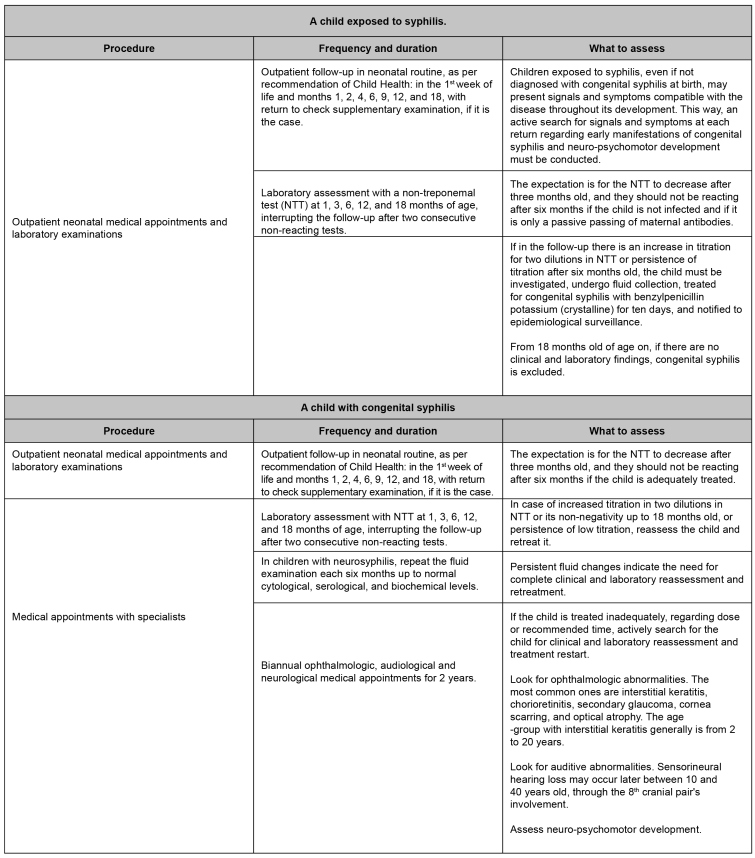
Clinical and laboratory follow-up of children exposed to syphilis and with congenital syphilis.

**Source:** adapted from Clinical Protocol and Therapeutic Guidelines for Comprehensive Care for People with Sexually Transmitted Infections, 2020^4^.

The maternity ward or birth house is responsible for referring, at the moment of discharge, all children exposed to syphilis and with congenital syphilis, treated or in treatment, to the healthcare units, preferably with a pre-scheduled appointment. Follow-up may be carried out during neonatal appointments in primary care service^36^, with surveillance and careful monitoring of signals and symptoms suggesting congenital syphilis, in addition to syphilis tests and supplementary examinations (Figure 5). We highlight that outpatient follow-up must be assured to all children exposed to syphilis or congenital syphilis up to 18 months old. It is essential to highlight that no mother or newborn must leave the maternity ward without knowing the syphilis test results during birth^11^. 

## SURVEILLANCE, PREVENTION, AND CONTROL

Brazil has high prenatal care coverage, although it is not uniform. According to the Information System on Live Births, the coverage of seven or more prenatal medical appointments among live newborns increased from 65%, in 2014, to 71% in 2018^37^. However, the number of medical appointments does not replace the quality of these appointments, as congenital syphilis is still a consequence of non-diagnosed or inadequately and untimely treated maternal syphilis. Actions for preventing congenital syphilis relate to pregnant women's care in the prenatal service, such as serological screening and the correct and timely treatment of maternal syphilis, established as soon as possible. The care efficiency provided to pregnant women requires compliance to all these parameters to avoid vertical transmission of *T. pallidum*.

Stimulating the father or partner’s participation throughout the entire prenatal care is very important for the mother’s biopsychosocial well-being, of the baby, and his own. It is crucial to implement male prenatal care and its treatment in case of syphilis or other STIs^38^ ^,^ ^39^. It should be stressed that, in addition to the stable partner or the child’s father, the pregnant woman may have other sex partners. Therefore, the healthcare team must be attentive and assist everyone with whom the pregnant woman is sexually associated.

Acquired syphilis, syphilis in pregnant women, and congenital syphilis are compulsorily notified diseases and must be notified in the appropriate form, sent to epidemiological surveillance^4^. It is essential to highlight that, despite the observed improvement, the notification of syphilis in pregnant women cases and its sex partners and congenital syphilis in public healthcare and supplementary services is still very incipient, which contributes to underestimating the occurrence of syphilis in Brazil. Prioritizing public policies encompassing sanitary authorities, healthcare managers, and the general population can lead to changes in the current syphilis scenario in Brazil.
